# Defective CFTR Expression and Function Are Detectable in Blood Monocytes: Development of a New Blood Test for Cystic Fibrosis

**DOI:** 10.1371/journal.pone.0022212

**Published:** 2011-07-21

**Authors:** Claudio Sorio, Mario Buffelli, Chiara Angiari, Michele Ettorre, Jan Johansson, Marzia Vezzalini, Laura Viviani, Mario Ricciardi, Genny Verzè, Baroukh Maurice Assael, Paola Melotti

**Affiliations:** 1 Sezione di Patologia Generale, Dipartimento di Patologia e Diagnostica, University of Verona, Verona, Italy; 2 Physiology Section, Dipartimento di Scienze Neurologiche, Neuropsicologiche Morfologiche e Motorie, University of Verona, Verona, Italy; 3 Centro Fibrosi Cistica, Azienda Ospedaliera Universitaria Integrata di Verona, Verona, Italy; 4 Sezione di Statistica Medica e Biometria “G.A. Maccacaro”, Dipartimento di Medicina del Lavoro “Clinica del Lavoro L. Devoto”, Università degli Studi di Milano, Milano, Italy; Comprehensive Pneumology Center, Germany

## Abstract

**Background:**

Evaluation of cystic fibrosis transmembrane conductance regulator (CFTR) functional activity to assess new therapies and define diagnosis of cystic fibrosis (CF) is cumbersome. It is known that leukocytes express detectable levels of CFTR but the molecule has not been characterized in these cells. In this study we aim at setting up and validating a blood test to evaluate CFTR expression and function in leukocytes.

**Description:**

Western blot, PCR, immunofluorescence and cell membrane depolarization analysis by single-cell fluorescence imaging, using the potential-sensitive DiSBAC_2_(3) probe were utilized. Expression of PKA phosphorylated, cell membrane-localized CFTR was detected in non-CF monocytes, being undetectable or present in truncated form in monocytes derived from CF patients presenting with nonsense mutations. CFTR agonist administration induced membrane depolarization in monocytes isolated from non-CF donors (31 subjects) and, to a lesser extent, obligate CFTR heterozygous carriers (HTZ: 15 subjects), but it failed in monocytes from CF patients (44 subjects). We propose an index, which values in CF patients are significantly (p<0.001) lower than in the other two groups. Nasal Potential Difference, measured in selected subjects had concordant results with monocytes assay (Kappa statistic 0.93, 95%CI: 0.80–1.00).

**Results and Significance:**

CFTR is detectable and is functional in human monocytes. We also showed that CFTR-associated activity can be evaluated in 5 ml of peripheral blood and devise an index potentially applicable for diagnostic purposes and both basic and translational research: from drug development to evaluation of functional outcomes in clinical trials.

## Introduction

Cystic fibrosis (CF) is primarily a disorder of electrolyte transport by epithelial cells in which an anion channel, activated by cyclic adenosine monophosphate (cAMP)-dependent kinase (the cystic fibrosis transmembrane conductance regulator-CFTR) is defective and represents the most common autosomal recessive disease with lethal consequences in Caucasians. Mutations in the *CFTR* gene result in aberrant variants that are either unstable, mis-localized, or with altered chloride conductance [1]. Although many antibodies have been developed against CFTR, only limited data describing CFTR expression in human leukocytes are available. In fact, most studies have focused on selected leukocytes or cell lines and provide only indirect measurements of CFTR [2], [3]. Functional CFTR is currently evaluated in humans using ex vivo and in vivo assays, the former method utilizing rectal biopsies [4]. This approach permits the direct recording of transepithelial currents (Intestinal Current Measurements, ICM) but requires an excision of a tissue sample. Nasal potential differences (NPD) [5] can be measured in vivo but require patient's collaboration, are time consuming and can only be performed in highly specialized centers Both these methods do not allow performing multiple assessments on the same subject within short intervals. Sweat test present normal/borderline results in some CF patients as previously described [6]. Moreover concomitant diseases or therapies could affect sweat tests [7].

To our best knowledge, only one blood test of potential clinical interest for the recognition of CF patients (using erythrocytes) has been proposed. However, this test has limitations in terms of sensitivity and specificity [8].

Recently, it has been reported that human alveolar macrophages express functional CFTR [9]. Our aim was to confirm and extend this finding by showing the expression of a specifically processed CFTR polypeptide in monocytes and to propose a functional assay using monocytes isolated from peripheral blood.

## Materials and Methods

### Subjects

Healthy volunteers including obligate heterozygote and individuals undergone genetic analysis for *CFTR* mutations for genetic counseling purposes were recruited for the study.

Peripheral blood was taken from 31 non-CF (median age 39.5 years, range 25–48 years; 12 males and 19 females), 15 heterozygous (HTZ: median age 44 years, range 4–67 years 4; 3 males and 12 females) and 44 CF subjects (median age 32 years, range 10–50; 23 males and 21 females). Samples and data were used for analysis only after that informed consent was obtained according to the guidelines approved by the local Ethical Committee. All clinical data were collected in the electronic database of the Cystic Fibrosis Center of Verona.

### Purification of monocytes from whole blood

Monocytes were obtained from 4–5 ml of fresh whole blood supplemented with 1 mM EDTA and purified using the RosetteSep® Human Monocyte Enrichment Cocktail (cat. # 15068 StemCell Technologies) according to the manufacturer's instructions. To remove platelets 20 µL of CD61 MicroBeads (cat. # 130-051-101 Miltenyi Biotec) was added and cells were incubated for 15 minutes at 4–8°C and treated according to the manufacturer's instructions.

### Cell lines

Cell lines utilized were the following: CFBE41o^−^, (kind gift from DC Gruenert, California Pacific Medical Center Research Institute, San Francisco, CA, USA) [10], 16HBE14o- (kindly provided by P. Davis, Case Western Reserve University School of Medicine, Cleveland, OH, USA) [11], the bronchial epithelial cell line IB3-1 derived from a CF patient with genotype W1282X/F508del (kind gift of Pamela Zeitlin, Johns Hopkins Hospital, Baltimore, USA) [12], pancreatic cell lines Suit-2 derived from a liver metastasis of a human pancreatic adenocarcinoma [13] and CFPAC-1 [14].

### Western blotting

Monocytes and epithelial cell lines were lysed with a solution containing: 150 mM NaCl, 50 mM TrisHCl, 1% (v/v) Triton X-100, 0.2 mM NaVO_4_, 1 mM DTT, 10 mM NaF, 1 mM EDTA and protease inhibitor cocktail (Roche, Milan, Italy). Proteins were quantified by Bradford method, denaturated in Laemmli sample buffer for 20 minutes at 40°C or 10 minutes at 95°C, then separated in SDS gel containing 6% acrylamide and subjected to western blotting onto nitrocellulose membranes (Millipore Corp., Bedford, MA). Membranes with transferred proteins were incubated over night at +4°C with or 0.3 µg/mL polyclonal anti-CFTR (ACL-006, Alomone Labs, Jerusalem, Israel) or 0.5 µg/mL of either of the monoclonal anti-CFTR antibodies clones 13-1 or 24-1 (R&D Systems, Minneapolis, U.S.A). After stripping, the same membranes were incubated with 0.2 µg/mL polyclonal anti-actin (Sigma-Aldrich, Missouri, U.S.A). In all cases detection of the primary antibody was performed with HRP conjugated secondary antibodies (GE Healthcare, Piscataway, U.S.A) followed by ECL detection (Millipore, Billerica, U.S.A).

### Immunofluorescence

Monocytes were purified as described above and seeded onto coverslips at a density of 450 cell/mm^2^. Cells were let to adhere for one hour and were then fixed for 10 minutes with 4% paraformaldehyde in PBS (BioLegend, San Diego, U.S.A). After blocking and permeabilization with 10% human serum and 0.1% (v/v) Triton®X-100 (both Sigma-Aldrich, Missouri, U.S.A) cells were incubated with 1 µg/mL monoclonal anti-CFTR antibody clone 13-1 (R&D Systems, Minneapolis, U.S.A) and then with 2 µg/mL Goat anti-Mouse IgG/AF594 (Invitrogen, Carlsbad, U.S.A). Nuclei were stained with 3 µM DAPI (Sigma-Aldrich, Missouri, U.S.A). Cells were analysed at Leica TCS-SP5 confocal microscope (Leica Microsystem, Wetzlar, Germany).

### Immune precipitation and in vitro phosphorylation of CFTR

30×10^6^ cells were washed twice with 50 ml ice cold TBS 1× and resuspended in 1 ml Hypotonic Buffer (10 mM HEPES-NaOH pH 7.9, 1.5 mM MgCl_2_, 10 mM KCl, 2 mM DTT, 1 mM PMSF) in the presence of protease inhibitor cocktail (Complete Mini, EDTA-free Roche Diagnostics, Mannheim, Germany) as previously described [15].

200 µg of light membrane preparation was precleared by adding 100 µl of suspended 20% v/v Protein G-Sepharose 4 Fast flow conjugate (PGS: GE Healthcare, Uppsala Sweden) and incubated at 4°C on a rotating wheel for 30 minutes. After centrifugation at 16,000× g for 30 seconds at 4°C, the supernatant was transferred, to a new ice-cold microcentrifuge tube containing 2 µg rabbit anti-CFTR (ACL-006 Alomone Labs Ltd., Jerusalem, Israel; 0.8 mg/ml) and 2 µg mouse anti-CFTR (MAB 3482 clone MM13-4 Chemicon International, Temecula, California USA; 1 mg/ml) or non-immune rabbit and mouse IgG and incubated for 2 hours at 4°C. Immune precipitation and in vitro phosphorylation of CFTR After centrifugation at 16,000× g for 10 minutes at 4°C, the resulting supernatant was transferred to a tube containing 10 µl of PGS and incubated at 4°C with rotation for 30 minutes. Beads were gently washed 3 times with 1 ml RIPA Buffer (150 mM NaCl, 1% Nonidet P-40, 0.5% Na deoxycholate, 0.1% SDS, 200 µM Na orthovanadate, 50 mM Tris-HCl pH 7.4, 1 mM DTT, 10 mM NaF, 1 mM EDTA) and once with 1 ml PKA Buffer (20 mM Tris-HCl pH 7.5, 0.5 mM DTT).

To phosphorylate CFTR, the pellet was resuspended in 50 µL PKA Buffer containing 250 ng PKA (PKA, His•Tag®, Active, Human, Recombinant, E. coli Calbiochem, Merck KGaA, Darmstadt, Germany; specific activity: ≥500,000 units/mg protein), 10 mM MgCl_2_, 10 µM ATP, 10 µCi γ^32^P-ATP (activity: 6000 Ci/nmol- 150 mCi/ml; Perkin-Elmer, Boston, MA USA) and incubated at room temperature for 30 minutes. After blocking the reaction with SDS-PAGE sample buffer, the sample was subjected to 6% SDS-PAGE. The gel was subsequently stained with Coomassie blue, dried and exposed to X-OMAT AR film.

### *CFTR* mRNA analysis by reverse-transcription and polymerase chain reaction (PCR)

Total RNA from monocytes purified as previously described was prepared using TRIzol extraction kit (Invitrogen, Life Technologies, Rockville, MD) according to the manufacturer's instructions. Primers used were: ACTB 170F, 5-ATCAAGATCATTGCTCCTCCTG-3; ACTB 170R, 5-GCAACTAAGTCA TAGTCCGCC-3; CFTR 771F 5-GGGGAAGTCACCAAAGCAGTACAGC-3; CFTR 771R 5-GCGCAGAACAATGCAGAATGAGATGG-3 [16]. These *CFTR* primers give a predicted product of 771 bp that was used as template for the following PCR, which was performed as above with the following primers (CFTR 338F 5-TCACATTGGAATGCAGATGAG-3; CFTR 338R 5-GTCTTTCACTGATCTTCCCA-3) expected to amplify a 338 bp product when exon 5 is present in the cDNA and of 248 bp if the exon 5 is skipped. Total RNA (1 µg) was reverse transcribed in a volume of 20 µl using 1 µM random primer (Invitrogen, Milan, Italy) and 200 U SuperScript II (Invitrogen, Milan, Italy) at 42°C for 1 hour, as described by the manufacturer. Polymerase chain reaction (PCR) was performed in a GeneAmp PCR System 9700 (PE Applied Biosystems, Milan, Italy) for 35 cycles (30 seconds of denaturation at 94°C, 30 seconds of annealing at 60°C, and 50 seconds of elongation at 72°C) in a volume of 25 mL reaction buffer containing 0.75 U AmpliTaq (PE Applied Biosystems), 0.4 M each primer, and 0.2 mM dNTPs (Roche, Milan, Italy). Actin mRNA amplification was performed for 22 cycles on the cDNA as positive control of reaction efficiency.

### Cell depolarization assay

The potential-sensitive probe bis- (1,3-diethylthiobarbituric acid) trimethine oxonol (DiSBAC_2_(3), Invitrogen, USA) was used to monitor CFTR-dependent membrane potential (Vm) changes in pancreatic adenocarcinoma cell lines derived from normal or CF subjects (Suit-2 and CFPAC-1 respectively) and in monocytes from non-CF, HTZ and CF patients. The DiSBAC_2_(3) method has already been described in detail [17], and was used with minor modifications. Before the assay, monocytes were sedimented (2×10^5^ cells/ml) onto a tissue culture dish (Falcon, Becton Dickinson, Franklin Lakes, NJ USA) for 1 h in RMPI 1640 medium supplemented with 1 mM L-glutamine at 37°C and then supplemented with medium containing 10% FBS. At the time of assay, 24 hours after preparation, the cells were washed twice with a Cl-free solution. Then, the cells were perfused for 10 minutes at room temperature with Cl-free solution containing 100 nM DiSBAC_2_(3). A baseline was acquired for 5 minutes before addition of a CFTR stimulus, consisting of a cocktail containing 500 µM 8-Br-cAMP (Sigma B5386), 10 µ Forskolin (Sigma F6886) and 100 µM 3-Isobutyl-1-methylxanthine (Sigma I7018). The CFTR inhibitor CFTR (inh)-172 (Sigma C2992) was used at a final concentration of 10 µM to specifically inhibit CFTR activity [18].

In some experiments amiloride (Sigma A7410) was added to a final concentration of 200 µM to block epithelial sodium channel.

The fluorescent signal was acquired on Zeiss microscope Olympus BXS1WI using a CCD intensified videocamera (Retiga EXi, Q-Imaging, Canada) and the software QED in vivo (MediaCybernetics, USA), at a rate of 1 frame/min. DiSBAC_2_(3) was excited with a 100-watt mercury-arc lamp and 535/40 excitation and 630/30 emission filters.

Data are presented as percentage of signal variation (ΔF) in relation to the time of addition of the stimulus, according to the equation: ΔF_t_ = [(F_t_-F_0_)/F_0_]×100, where F_t_ and F_0_ are the fluorescence values in the absence of extracellular Cl- at time t and at the time of addition of the stimulus, respectively.

### Transepithelial NPD measurements

NPD measurements were performed following the standardized procedure [19] consistent with the recommendations provided by the SOP and applied following the procedures set with the multicenter basic protocol [20]. In particular, the potential difference (PD) was determined by measuring the PD between a Ringers-filled exploring catheter on the nasal mucosa and a reference bridge (21-gauge needle filled with Ringers solution) inserted into the subcutaneous space of the forearm. PE50 tubing (Fisher Scientific BD) was utilized as catheters. Each nostril was examined using an otoscope. The inferior turbinate was explored with a catheter for the site of most negative voltage; the probe was positioned with tape on the nose and forehead. Baseline PD was determined by perfusion with Ringers and the responses to amiloride (1×10^−4^ M), zero chloride (0 Cl^−^), isoproterenol (1×10^−5^ M) and ATP (1×10^−4^ M) were sequentially acquired for a minimum of 3 minutes each obtaining a period of stable signal for at least 30 sec. We perfused solutions warmed to 37°C by passing the polyethylene tubing through a water-jacketed tube proximal to the nasal probe in order to obtain a larger activated chloride conductance [21]. The software PowerLab (ADInstruments, UK) was utilized for acquisition and analysis of data. The data acquisition system ML870 PowerLab 8/30 was utilized in connection with the voltmeter Iso Millivolt Meter (World Precision Instruments, Sarasota (WPI), FL, USA). It was connected to Ag/AgCl electrodes MEH3S (World Precision Instruments, FL, USA) linked to the catheters.

Smokers and subjects with nasal polyps or abnormal mucosa were not subjected to this test.

### Analysis of cell depolarization assay data

Before analysis, data were transformed to control for bias potentially generated by experimental setting variation: fluorescence was expressed as % variation between time t and time 0 according to the formula: ΔF_t_ = [(F_t_-F_0_)/F_0_]×100. The first 5 time points were excluded from the analysis due to the perturbation of the system as a consequence to the addition of compounds that required the system to reach equilibrium before starting the recording.

The % variation of fluorescence over time and 3 phenotypic groups (CF, non-CF and HTZ) and their interaction were fitted with a mixed linear model including individual response curves as random term.

Q-Q plots of residuals and plots of residuals against predicted values were inspected to check whether model assumptions were met.

We propose a summary measure of the ΔFt curves, computed as difference between the mean ΔFt (stimulus), and the mean ΔFt (vehicle) in the last five minutes of recording, as described. We termed this value “CF index”.

To test whether CF index values differ between phenotypic groups or between classes of CFTR mutation, analysis of variance was performed on normal scores computed on CF-index ranks [22]. Statistical significance was set at the level of P<0.05. Bonferroni procedure [23] was used to adjust p-values when multiple tests were carried out. Analyses were performed with SAS 9.2 (SAS Institute, Inc., Cary, NC).

## Results

### CFTR expression in epithelial cells and monocytes

**Figure 1:**
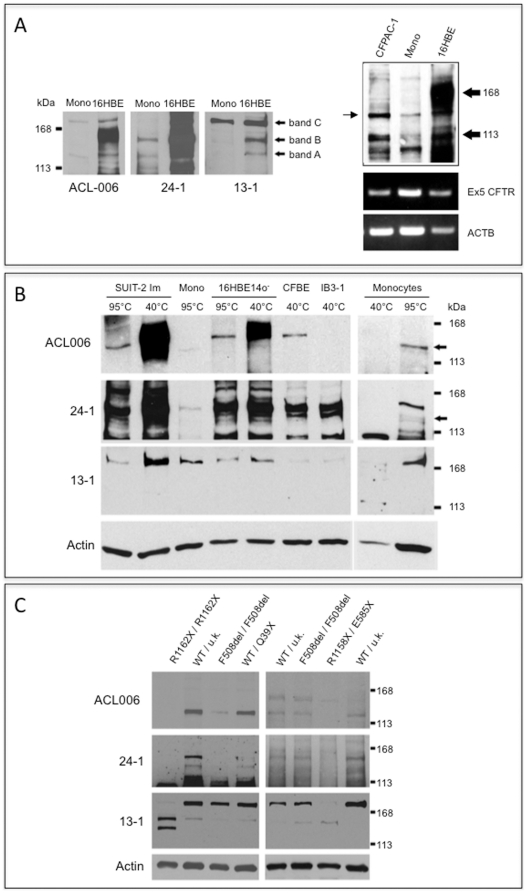
Expression of CFTR in epithelial cells and monocytes. **Panel A**: Monocyte cell lysates were denatured at 95°C while epithelial cells were treated at 40°C before SDS-PAGE. The left panel shows the result after the incubation with the anti- CFTR antibody ACL-006, 24-1 or 13-1. Although in epithelial cells all the CFTR forms (A, B and C) are detected (although with a different pattern of reactivity depending on the type of antibody), in monocytes not all the bands are detected by the individual antibodies, although all of them are present as testified by the recognition of bands A, B and C by their combination. While band A is consistently detected by ACL-006 antibody in monocytes, detection of the other bands with this antibody is not reproducible in all the samples tested. On the right side of the panel the arrow indicates the less glycosylated form of CFTR (band A) expressed in 16HBE14o- cells, CFPAC-1 and monocytes (ACL-006 antibody). mRNA of CFTR as detected by RT-PCR is expressed by monocytes that express the same spliced isoform expressed by epithelial cells. Actin (ACTB) has been amplified as control. **Panel B**: Temperature-dependent detection of CFTR Effect of the denaturating temperature on the CFTR bands detected in epithelial cells and monocytes. A major band around 170 kDa (band C) is detected in both epithelial cells lines expressing wild-type CFTR (Suit 2 and 16HBE14o-) and in monocytes (13-1 antibody). CFBE41o^−^ (CFBE) (F508del/F508del) cells express a single band corresponding to a core-glycosylated CFTR (band B, ACL-006 and 24-1 antibodies) while CFTR expression level in IB3-1 (W1282X/F508del) is below the detection limit of the assay for ACL-006 antibody but display a band using the 24-1 antibody. The incubation with ACL-006, 24-1 and 13-1 antibodies, reactive against a C- and a more N-terminal epitope (13-1), when analyzed together, identify all the glycosylated forms in our assay only when the most appropriate temperature is utilized (40°C for epithelial cells and 95°C for monocytes). Of note is the loss of CFTR signal in monocytes treated at 40°C associated to a reduction of immunoreactivity of anti-actin antibody used to demonstrate equal protein load (last two lanes, right). **Panel C**: Western blotting with the indicated antibodies on lysates of monocytes derived from patients with the indicated genotypes. Note that the bands corresponding to a full length CFTR present in wild-type (WT) or obligate heterozygotes (WT/Q39X) are missing in monocytes derived from patients homozygous for nonsense mutations (13-1 antibody). Patients carrying one or two F508del alleles express a much fainter band. Actin expression is shown to demonstrate equal protein load.

Western blotting analysis of cell lysates from pancreatic and bronchial epithelial cell lines expressing wild-type CFTR (Suit-2 and 16HBE14o-) allows to identify the presence of all the major CFTR bands described in the literature, namely bands A, B and C (**Figure 1A**). The CFTR isoform A corresponds to a MW of approximately 130–140 kDa corresponding to an immature, incompletely-glycosylated form of CFTR as shown in 16HBE14o- and CFPAC-1 cell lines [24] (Figure 1, panels A and B). Another isoform (isoform B) is also typical of the incompletely glycosylated (“core glycosylated”) CFTR expressed by F508del/F508delexpressing cells as shown for CFBE41o^−^ while the fully glycosylated form (C) is also detectable (**Figure 1** **, panel B**). The apparent MW of CFTR expressed by CF pancreatic epithelial cell line CFPAC-1 corresponds to that of band A expressed by 16HBE14o- and by human monocytes as detected by ACL-006 antibody (**Figure 1** **, panel A**).

All three antibodies utilized recognize different epitopes present in all CFTR forms expressed in epithelial cells but present with a slightly different pattern, likely based also on the type and extent of post-translational processing. CFTR expressed in monocytes is present in all the known forms (A, B and C), however none of the individual antibodies appear capable to recognize all the forms expressed by monocytes as it occurs (albeit at different extent) in epithelial cells. Indeed ACL-006 antibody detects in monocytes bands A and (inconstantly) C, 24-1 bands A and B while 13-1 recognize band C. When the data are combined, all the differentially processed forms of CFTR appear to be expressed by monocytes. Of note, CFTR is usually detected in epithelial cells upon treatment of the cell lysates at room temperature or 40°C [25]. We did confirm these observations in epithelial cells (Figure 1 panel B), however we discovered that CFTR expressed in monocytes is detectable only when cell lysates are treated at 95°C. Treatment at 40°C apparently induces protein degradation as suggested by the reduced expression of actin in the same lysates incubated in reducing sample buffer at 40°C in comparison with the same sample treated at 95°C where complete protein denaturation (and protease inactivation) are known to occur (**Figure 1** **, panel B**, last two lanes).

The monocytes express the same mRNA form detectable in epithelial cells as demonstrated by RT-PCR on the same cells analyzed by western blotting with primers located on exons 4 and 6. These primers amplify the region containing exon 5, known to be excluded in a 140 kDa partially functional CFTR isoform detected in human cardiac muscle [26] (**Figure 1** **, panel B**).

The specificity of the signal detected with the antibodies is further strengthened by the observation of the loss of the C-terminal epitope in monocytes derived from patients carrying two nonsense mutations (R1158X/E585X; R1162X/R1162X). CFTR expression is also severely impaired in monocytes derived from patients with F508del/F508del genotypes as expected (**Figure 1** **, panel C**).

**Figure 2:**
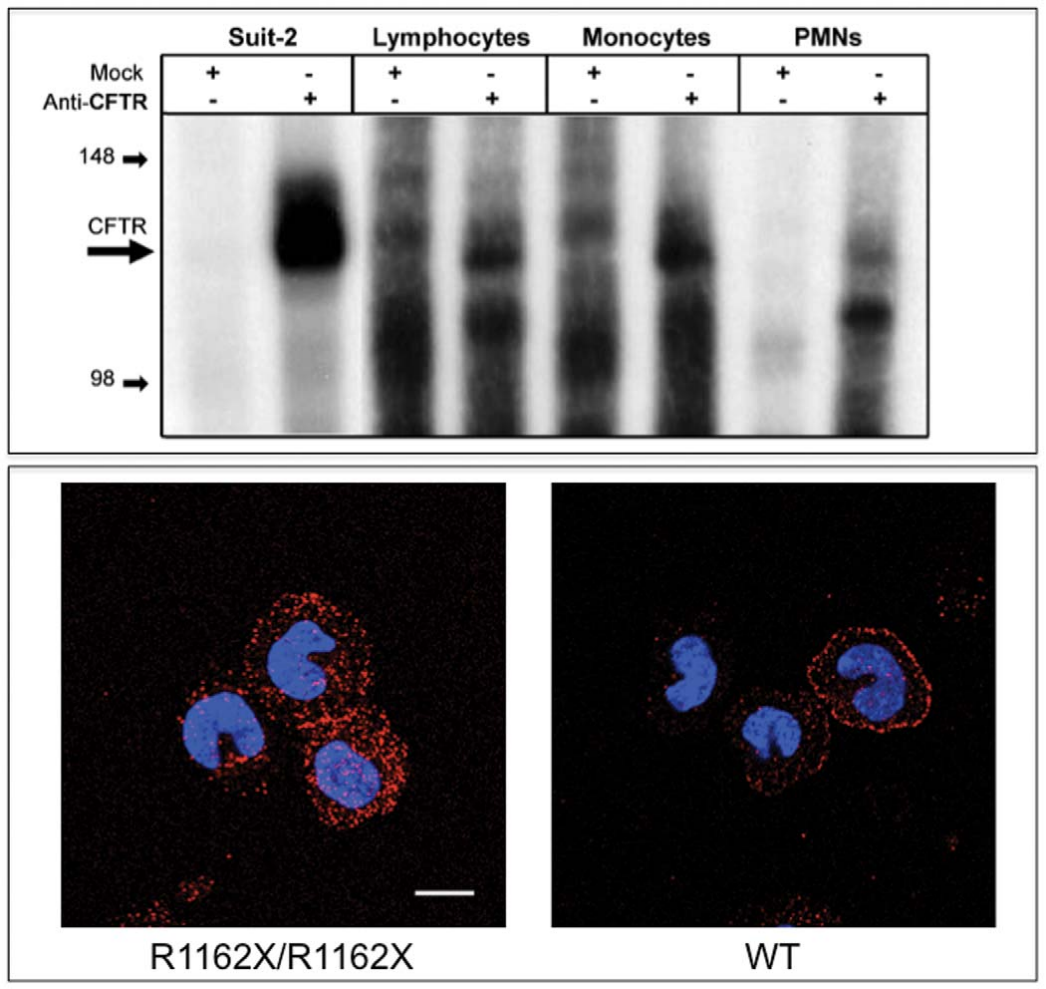
Expression of CFTR in leukocytes. **Upper Panel**: Membrane localization of CFTR in leukocytes. CFTR was immune-purified from 200 µg of light membrane samples of non-CF lymphocytes, monocytes and neutrophils using a mixture of two anti-CFTR antibodies (ACL-006 and MM13-4) and subjected to PKA-dependent in vitro phosphorylation. CFTR was detected as a specific band at approximately 140 kDa. The Suit-2 pancreatic adenocarcinoma cell line was used as a positive control. The band was not detectable when immune precipitation was performed with isotypic antibodies (Mock). **Lower Panel**: Confocal microscopy analysis performed using the 13-1 antibody on monocytes derived from subjects carrying a truncated (R1162X/R1162X) and wild type forms of CFTR. Membrane localization is detected in WT monocytes while R1162X/R1162X ones display a disorganized distribution of the signal. The bar indicates 10 µM (one experiment of 3 performed).

We next wondered whether CFTR was properly expressed on the plasma membrane of monocytes. To answer to this point we applied the combination of a biochemical assay with confocal microscopy analysis. The immunoprecipitated CFTR protein expressed in peripheral blood leukocytes (monocytes, lymphocytes and, albeit at lower levels, granulocytes) is detectable in the membrane fraction where it represents an in vitro substrate for the catalytic subunit of protein kinase A (**Figure 2** **, panel A**).

The membrane localization of CFTR expressed by non-CF monocytes is confirmed by confocal microscopy analysis (Figure 2, bottom right panel), where non-CF monocytes expressing membrane-localized CFTR are shown in comparison with the monocytes isolated from patients carrying a R1162X/R1162X, predicted to produce a truncated form of the protein as confirmed by western blotting data (**Figure 1** **, panel C**) that appears to loose membrane localization, as detected by confocal microscopy analysis (Figure 2, bottom left panel).

Altogether these data indicate that monocytes express a CFTR polypeptide with biochemical features slightly different from those expressed by epithelial cells, but possessing all the features deemed necessary for a functional channel [2], [9], [24].

### CFTR functional analysis in epithelial cell lines and primary monocytes by single cell fluorescence analysis

**Figure 3:**
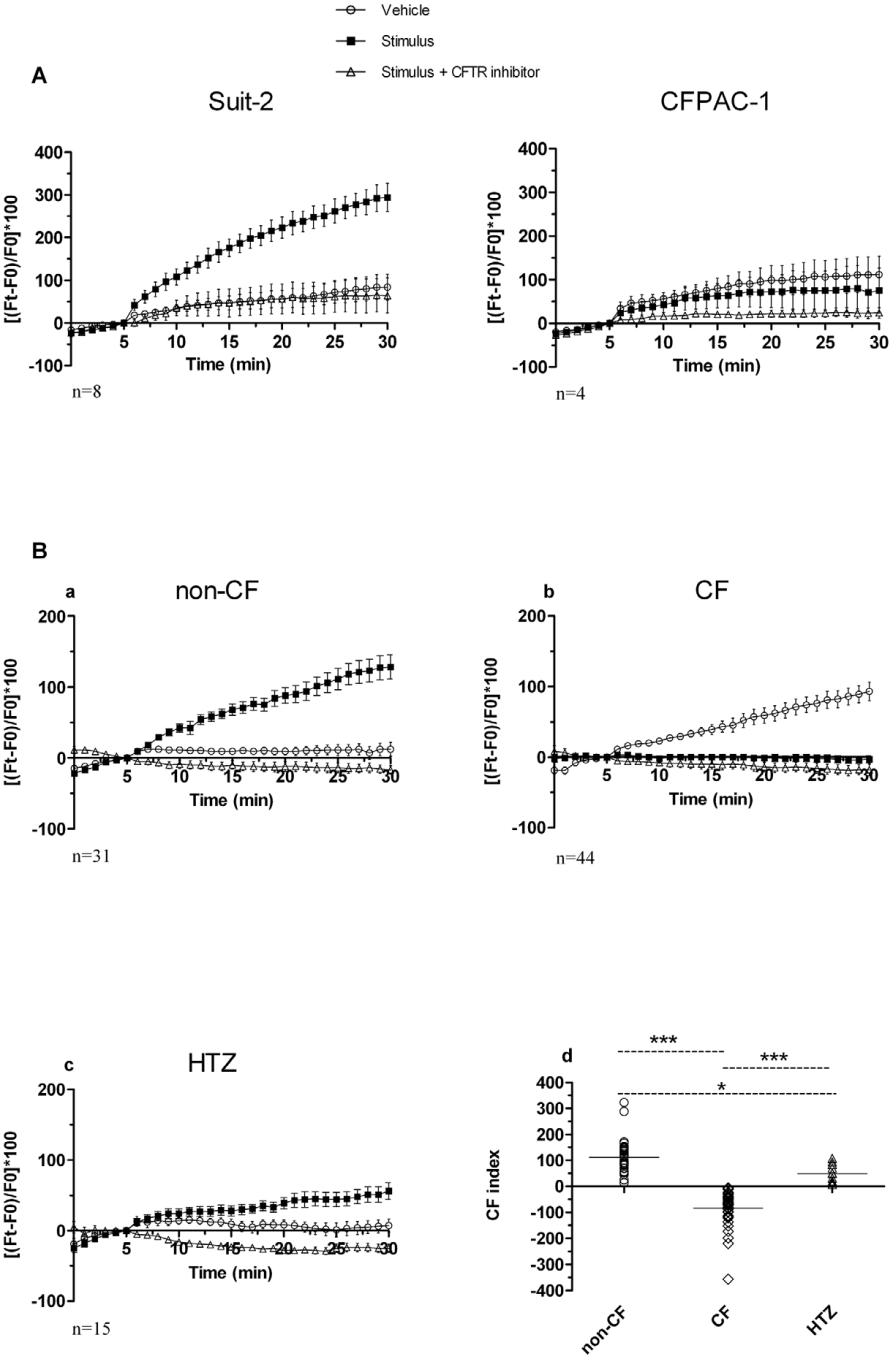
Fluorescence-based CFTR functional analysis. Percentage of DiSBAC_2_(3) fluorescence variation (ΔF) is shown over time of addition of the stimulus 8-Br-cAMP/forskolin/IBMX, according to the equation: ΔF_t_ = [(F_t_-F_0_)/F_0_]×100, where F_t_ and F_0_ are the fluorescence values in absence of extracellular chloride at time t and at the time of addition of the stimulus respectively. Graphs show mean ΔF_t_ and standard error. CFTR (inh)-172 (CFTR inhibitor) was added where indicated. **Panel A**: Suit-2 and CFPAC-1 pancreatic adenocarcinoma cell lines used as positive and negative controls, respectively, at the recording times indicated on the x axis. **Panel B**: Same as panel A using purified human monocytes of non-CF (a), CF (b) and HTZ (c) donors. (d) Differences between the maximum fluorescence value obtained under stimulation and the matching fluorescence value of vehicle (CF-index). CF group showed values of CF-index significantly lower (median: −70.2, IQR: −113.7;−47.5) both than the HTZ group (median: 50.5; IQR: 17.8, 84.7) and the non-CF group (median: 113.3, IQR: 57.3, 147.3), both p<0.001 (***); also the difference found between the non-CF and HTZ groups was statistically significant (p = 0.015, *).

To evaluate the functional activity of CFTR expressed in leukocytes we applied a method based on membrane potential (Vm) changes in single-cell, detected by the fluorescent probe bis- (1,3-diethylthiobarbituric acid) trimethine oxonol (DiSBAC_2_(3)), a member of the slow dye family [17]. The fluorescence quantum efficiency of DiSBAC_2_(3) increases when the dye moves from aqueous solution into membranes. A change in Vm, as for example during a depolarization, results in additional transfer of the dye into membranes, thus increasing the fluorescent signal. The experimental conditions for the CFTR functional assay were established using the Suit-2 and CFPAC-1 pancreatic cancer cell lines by measuring the membrane depolarization induced by treatment with the cAMP analog 8-Br-cAMP (stimulus), control baseline was recorded with the addition of vehicle alone (vehicle). 8-Br-cAMP induced a rapid fluorescence increase in Suit-2 cells, that was almost completely inhibited by treatment with the CFTR inhibitor CFTR (inh)-172 [18], while in CFPAC-1, a pancreatic adenocarcinoma cell line derived from a patient affected by CF (F508del/F508del genotype) cAMP analog was not able to induce fluorescence changes (**Figure 3A**).

**Figure 4:**
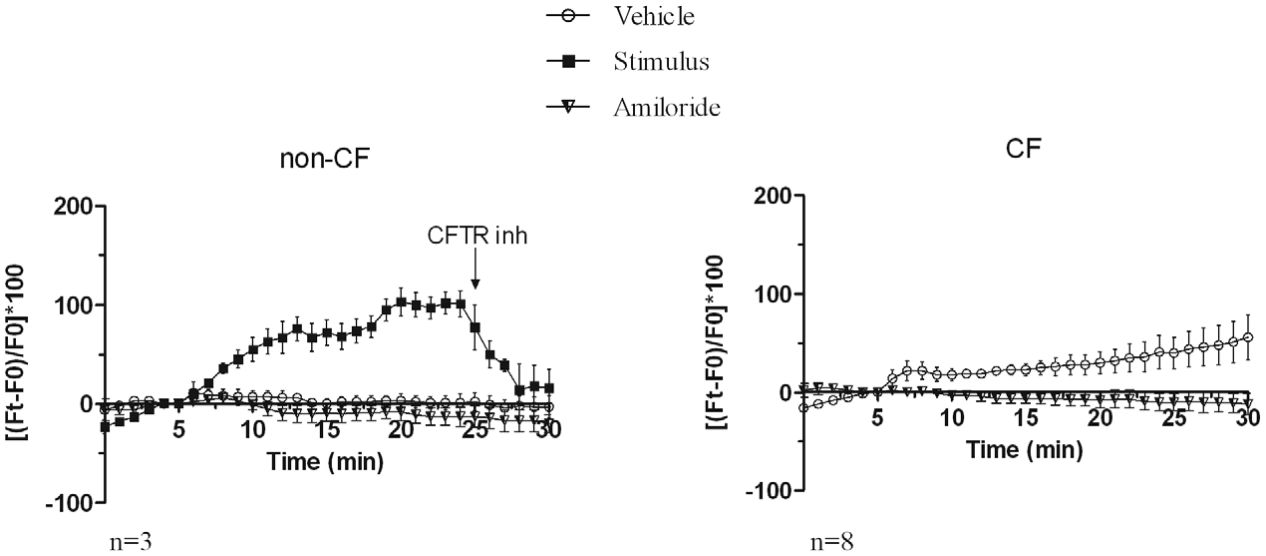
Amiloride inhibits the spontaneous depolarization signal detected in CF monocytes. Non-CF monocytes were analyzed with vehicle alone and in the presence of amiloride (left panel). Amiloride does not affect baseline. Amiloride treatment induces a reduction of spontaneous depolarization in CF subjects (right panel) to an extent overlapping to the effect of the addition of the stimulus. The effect of CFTR (inh)-172 after 15 minutes after addition of the stimulus in non-CF monocytes is also shown (left).

In non-CF monocytes the stimulus induced a membrane depolarization that was completely reversed by CFTR (inh)-172 (**Figure 3B, a**). In the cells derived from HTZ subjects, the increase in fluorescence following stimulation was substantially lower than that detected in non-CF (**Figure 3B, c**). Monocytes isolated from CF subjects (listed in **Table 1**) responded to stimulation with a decrease of the baseline fluorescence signal, and thus the response was below the baseline of spontaneous depolarization indicating a defect in the membrane conductance (**Figure 3B, b**). This effect was highly reproducible and was sensitive to the sodium channels blocker amiloride (**Figure 4**), suggesting their involvement in this phenomenon.

**Table 1:** Clinical information and laboratory data of the CF patients enrolled in the study.

Case	Gender	Age at diagnosis (years)	*CFTR* genotype*		Age (years)	Sweat Cl-mEq/L**	FEV1 % mean values 2009	Pa	PI	NPD results***	CF-index
1	F	0	3132delTG	1497delGG	34	129	75	yes	yes	nd	−22,10
2	F	0	R1162X	R1162X	43	144	52	yes	yes	nd	−29,65
3	M	0	R1162X	R1162X	10	102	59	no	yes	1,02	−10,18
4	M	0	R1162X	R1162X	25	115	81	no	yes	1,07	−67,11
5	M	7	G542X	711+5 G>A	24	105	59	yes	yes	nd	−5,84
6	M	1	CFTRdele1	G542X	36	107	22	yes	yes	nd	−113,92
7	M	0	G542X	G542X	16	110	71	yes	yes	0,97	−80,20
8	F	1	Q552X	CFTRdele17a-18	35	99	72	yes	yes	2,08	−219,81
9	M	16	R1162X	3849+10 Kb C>T	42	74	43	yes	no	1,02	−71,47
10	M	0	R1162X	R1162X	32	105	45	yes	yes	1,43	−114,67
11	M	1	F508del	F508del	16	86	71	no	yes	nd	−60,04
12	F	0	F508del	F508del	16	88	118	no	yes	nd	−48,20
13	M	0	F508del	F508del	33	118	51	yes	yes	nd	−65,49
14	M	7	F508del	F508del	37	89	37	yes	yes	nd	−359,82
15	F	0	F508del	F508del	27	118	71	yes	yes	nd	−67,26
16	F	8	1717-1 G>A	F508del	38	140	74	yes	yes	nd	−136,80
17	F	0	R1158X	F508del	32	95	60	yes	yes	1,77	−28,31
18	M	7	G542X	F508del	39	110	46	yes	yes	nd	−47,52
19	M	0	Q39X	F508del	17	101	79	no	yes	1,11	−64,20
20	F	1	R1162X	F508del	41	188	60	no	yes	0,94	−96,73
21	M	13	3849+10 Kb C>T	F508del	24	76	78	yes	no	4,67	−6,33
22	M	0	W1282X	621+1G>T	33	119	77	yes	yes	1,27	−42,74
23	F	4	R553X	2789+5 G>A	31	92	44	yes	no	7,4	−60,94
24	F	11	F508del	R553X	39	116	55	yes	yes	nd	−113,67
25	M	12	F508del	3849+10 Kb C>T	27	51	71	yes	no	1,12	−98,84
26	F	0	F508del	G542X	19	109	109	yes	yes	nd	−173,24
27	F	0	F508del	R1162X	32	94	86	yes	yes	1,34	−70,16
28	F	0	F508del	W57X (TAG)	27	99	78	yes	yes	1,21	−69,33
29	M	0	F508del	Q552X	24	94	41	yes	yes	1,50	−72,75
30	M	20	F508del	3849+10 Kb C>T	43	58	60	no	no	1,13	−112,56
31	M	0	F508del	R1162X	12	99	65	no	yes	2,14	−80,92
32	M	4	F508del	3849+10 Kb C>T	17	60	100	no	no	nd	−121,31
33	F	1	F508del	1717-1 G>A	26	105	73	yes	yes	2,05	−55,66
34	F	11	F508del	3849+10 Kb C>T	40	85	59	yes	no	nd	−152,23
35	F	4	F508del	1717-1 G>A	44	130	97	yes	yes	nd	−116,56
36	M	13	F508del	3849+10 Kb C>T	43	70	65	yes	no	CF	−65,10
37	F	19	F508del	unknown	29	95	100	no	no	nd	−40,53
38	M	6	F508del	unknown	15	92	87	yes	no	nd	−70,17
39	F	0	G542X	N1303K	34	108	97	yes	yes	nd	−96,14
40	M	50	G1249R	IVS8 T5TG12	50	61	74	no	no	nd	−199,15
41	F	10	2183 AA>G	IVS8 T5TG15/T7TG10	45	79	29	yes	no	1,9	−86,27
42	F	1	G85E	unknown	43	120	107	yes	no	nd	−49,21
43	F	0	3272-26 A>G	I507del	21	113	88	no	no	nd	−36,79
44	M	8	F508del	D1152H	10	77	107	no	no	nd	−10,85

*Cystic Fibrosis mutation database reference: http://www3.genet.sickkids.on.ca/cftr/app.

**As determined by Sweat test performed according to the Gibson and Cooke method (Sweat testing: Sample Collection and Quantitative Analysis – Approved Guideline – Second Edition – The National Committee for Clinical Laboratory Standards (NCCLS) – document C34 A2 [ISBN – 56238 – 407 – 4]; USA . Pa: Pseudomonas Aeruginosa chronic infection according to Doring et al [44]; PI (pancreatic insufficiency requiring oral enzyme supplementation); NPD: Nasal Potential Difference; nd: not done; CF: cystic fibrosis.

***Numbers indicate NPD values calculated as described by Wilschanski et al. [27].

### Analysis of fluorescence over time by phenotypic group and proposal of an index (CF-index) to differentiate non-CF, HTZ and CF subjects

As **figure 3B** (panels a–c) shows, ΔFt (vehicle) increases over time in the CF group, whereas it remains almost unchanged in the non-CF and HTZ groups. Conversely, ΔFt (stimulus) does not change over time in the CF group, taking values around zero and constantly increases over time in the non-CF group. Interestingly, the ΔFt (stimulus) curve of HTZ group runs between the CF and the non-CF group.

The differences ΔFt (stimulus) – ΔFt (vehicle) was calculated and the results were named “CF-index”. Statistical analysis of the data performed in the three groups confirmed the presence of a decreasing trend over time in this difference in the CF group (p<0.0001) and the increasing trend in the non-CF (p<0.0001) and in the HTZ (p = 0.04) groups. The analysis further confirmed the difference in the ΔFt (stimulus) – ΔFt (vehicle) curve between the groups CF and non-CF (p<0.0001), between the CF and HTZ (p<0.0001) and the non-CF and HTZ (p = 0.02) groups.

**Figure 3B** (panel d) shows the distribution of CF-index values in the three phenotypic groups. CF group showed values of CF-index significantly lower (median: −70.2, IQR: −113.7, −47.5) both than the HTZ group (median: 50.5; IQR: 17.8, 84.7) and the non-CF group (median: 113.3, IQR: 57.3, 147.3), both p<0.001; also the difference found between the non-CF and HTZ groups was statistically significant (p = 0.015).

However, the ROC curve computed on CF-index values for HTZ and non-CF participants showed poor ability of CF-index to discriminate between these groups (**Figure S1**).

The CFTR inhibitor 172 inhibits the depolarization induced by stimulation of non-CF monocytes (**Figure 4**).

**Figure 5:**
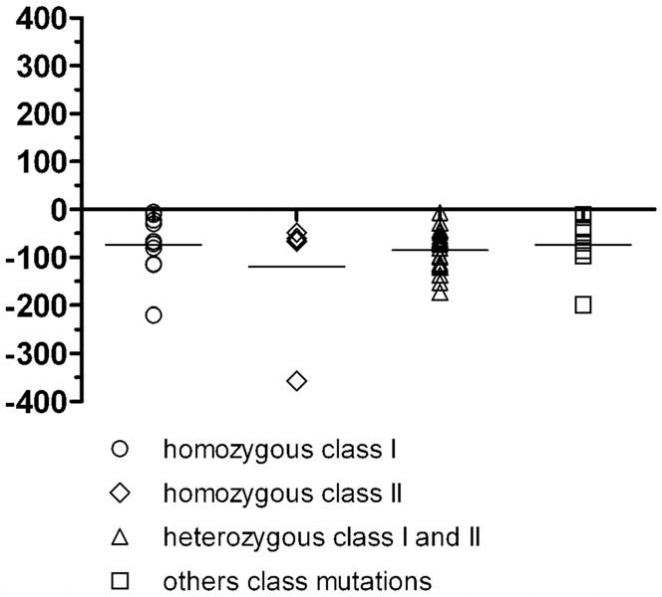
Distribution of CF-index by group of CFTR mutation. The CF subjects were categorized into 4 groups according to CFTR mutation. The five classes of CFTR mutation as described by Welsh and Smith [42] were adopted for this study according to the information available in the CFTR mutations database (http://www.genet.sickkids.on.ca/cftr/app). No significant differences were found between the mean values of CF-index in the 4 different genotype groups (homozygous class I, homozygous II, heterozygous class I and II and the other class mutations) on the basis of one-way ANOVA performed on normal scores computed on CF-index ranks.

To ascertain whether *CFTR* mutations have an impact on the values of the CF-index, we classified the mutations of CF patients into classes. Analysis of variance revealed that there is no evidence of association between the values of CF-index and *CFTR* mutation class (p = 0.79), as shown in **Figure 5**. Interpretation of data did not change considering other time points after at least 15 min of recording instead of the last 5 min (data not shown).

### Comparison among NPD measurements and monocyte test

We wanted to evaluate whether the results of the monocyte tests could identify correctly the patients found positive to an established diagnostic test measuring CFTR-dependent activity like NPD, a CFTR functional test based on the recording of Vm on epithelial cells of the upper respiratory tract. This test, unlike the monocyte test, is performed in vivo in the nostrils of collaborative individuals and requires that the patients are free of diseases affecting the test area. We evaluated the correlation between the NPD test results and the CF-index in 32 participants (20 CF, 4 HTZ and 8 controls) for whom a blood sample was obtained at the time of the NPD test. In order to be able to perform this analysis we calculated the Wilschanski's index that permits to evaluate the results of NPD measurements [27].

The comparison between Wilschanski's and CF-indexes shows that there are two outliers for which the value of the Wilschanski's index is particularly high consistently with the responses to low-Cl and isoprotenerol obtained in NPD tracings (**Figure S2**).

To reduce the influence of the two outliers, and due to the distribution of the Wilschanski's index, we decided to compute the Spearman rank-order correlation coefficient, which resulted equal to −0.57 (p = 0.0007). However, the scatter plot casts some doubts on the use of the correlation coefficient to analyze such data. In particular, the graph shows a clear separation in points: in the upper-left quadrant defined by CF-index>0 and Wilschanski's index <0.85 lay all the controls, whereas in the bottom-right quadrant lay all the CF participants. We therefore preferred to express the relation between our test and the NPD test in terms of concordance rather than correlation. We dichotomized the results, using zero as positivity threshold for the CF-index (as suggested by the plot of the index within groups) and we used 0.85 as positivity threshold for the Wilschanski's index (according to the validation of this index in groups of CF and non-CF subjects tested in our Center).

There was excellent concordance (Kappa statistic 0.93, 95%CI: 0.80–1.00) between the two test results: all the subjects were classified in the same category except one (HTZ group), that according to the index of Wilschanski was pathologic (index value = 1.68), but according to the CF-index it was not (index value = 94.2). Therefore the proportion of positive agreement [28] was 97.6% and the proportion of negative agreement was 95.6%.

**Figure 6:**
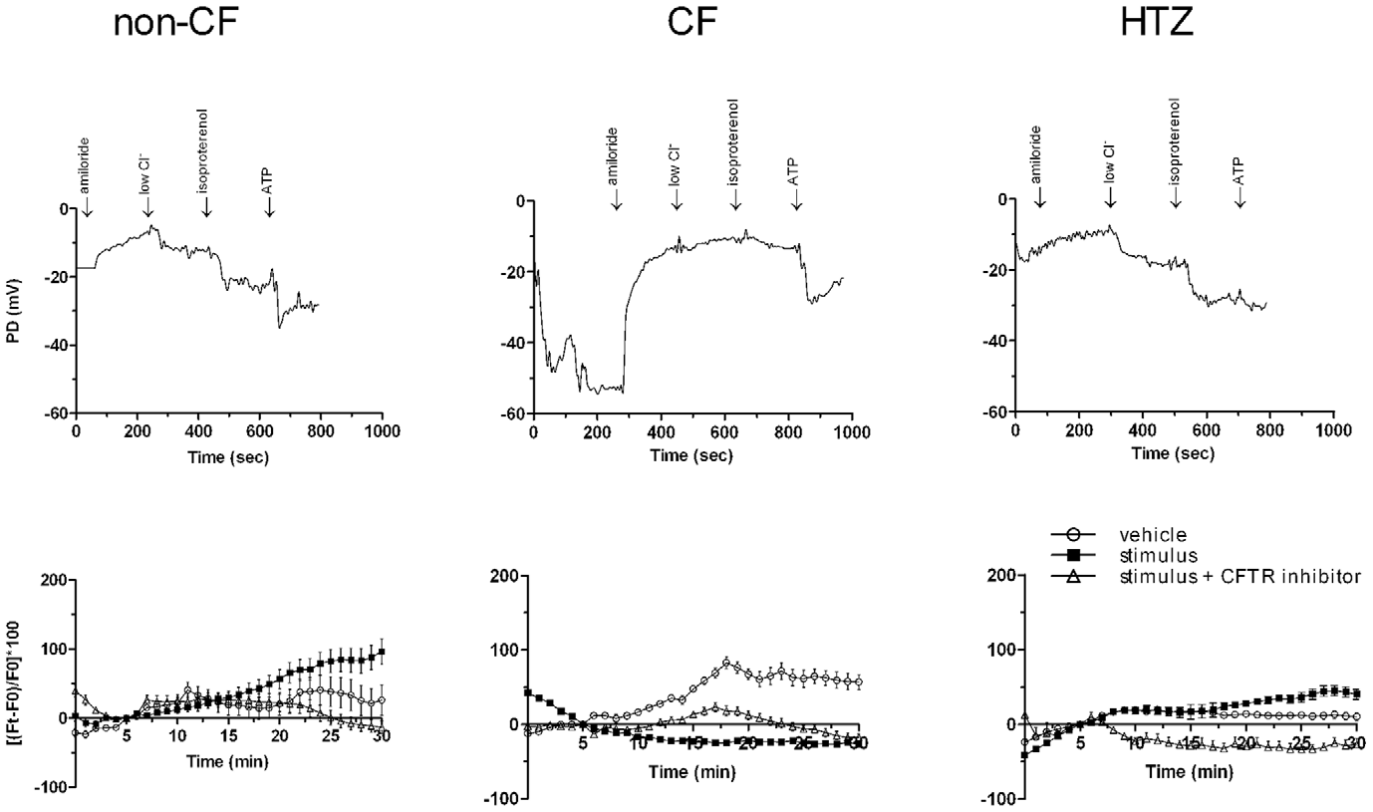
Nasal potential difference analysis (NPD) confirms the results obtained in monocytes isolated from the same subjects. Typical NPD measurements are shown in the upper panel: the agents indicated at the bottom were added at the times (minutes) shown on the x axis. On the y axis, PD is expressed in mV. The graphs represent a typical value obtained from the analysis of both nostrils in each subject. The presence of functional CFTR in non-CF and HTZ subjects is indicated by the marked response to the absence of chloride and the addition of isoproterenol, which is undetectable in CF subjects. Moreover, depolarization in the presence of amiloride is more consistent in CF than in non-CF subjects, as the cellular influx of sodium is increased in CF versus non-CF. Depolarization in the presence of amiloride is higher in CF than in non-CF and HTZ subjects in keeping with the increased cellular sodium influx in CF versus non-CF. Increased polarization is induced by no chloride and isoproterenol in the presence of functional CFTR in non-CF and HTZ subjects, but not in the presence of defective CFTR, as in CF subjects. ATP has been utilized as a stimulus for alternative non-CFTR chloride secretory pathways as Ca^++^ activated chloride channels [43]. The monocytes of the same subjects were tested, and the results are shown as indicated in B. Fluorescence analysis in monocytes shows that induction of depolarization was detected in non-CF and, to a lesser extent, in HTZ subjects but not in CF patients.

**Table 1** summarizes the clinical and laboratory data of patients, including the subjects that performed NPD test and **Figure 6** shows an example of the results obtained. We concluded that the monocyte test could differentiate CF from control subjects as the NPD test does.

## Discussion

In this study, we have characterized the expression of CFTR in human monocytes and described for the first time that these cells express a CFTR variant that is recognized by specific antibodies only following high-temperature denaturation. Moreover, we have also shown that the functional evaluation of CFTR in these cells is a potentially powerful tool for basic-translational research and might find diagnostic applications. Monocytes appear to express a CFTR corresponding to all the forms (defined as bands A, B and C in the literature) detected in epithelial bronchial cells. In order to detect CFTR expression in monocytes, cell lysate needs to be treated at 95°C while denaturation at 40°C is necessary to detect the antigen in epithelial cells. The same (95°C) treatment performed in epithelial cells leads to an almost complete loss of immuno-reactivity, with the notable exception of an incompletely glycosylated isoform roughly corresponding to the apparent molecule weight of band B. These results suggest that CFTR processing is both quantitatively and qualitatively different in monocytes as compared to both pancreatic and bronchial epithelial cells.

The specificity of the result is further confirmed by the loss of the specific band detected in normal monocytes in patients homozygous for nonsense mutations known to affect CFTR protein expression. Two CF patients heterozygous for nonsense and homozygous for F508del mutations showed a severely impaired expression of the band, in accordance with the predicted phenotype.

Furthermore, despite the fact that the apparent MW recorded is identical to that observed in WT and CF epithelial cells, we have also excluded the possibility that an alternatively spliced isoform missing exon 5 (30 amino acid) could be expressed in monocytes, as reported for cardiac muscle where only a core glycosylated, partially functional variant of about 140 kDa is expressed [26]. Accordingly RT-PCR performed using primers located in sequences located in exons 4 and 6 amplified a cDNA fragment predicted for the presence of exon 5 in both epithelial cells and monocytes. This test also confirms the presence of CFTR transcript in monocytes.

Whether this CFTR isoform expressed in monocytes is functional has been confirmed by its proper membrane localization (as detected by in vitro phosphorylation assay and confocal microscopy analysis) and by single-cell membrane depolarization analysis. This last assay confirms that, upon stimulation with CFTR agonists, normal monocytes display a highly reproducible membrane depolarization activity consistent with the expression of a fully functional CFTR. This response is inhibited by treatment with a specific CFTR inhibitor, is lost in monocytes derived from individuals affected by CF and is reduced in HTZ subjects. The spontaneous depolarization detected in monocytes derived from CF patients was an unexpected finding and allows us to design an index based on the differences recorded among the signals detected in the various experimental conditions. The spontaneous depolarization is inhibited by treatment with the sodium channels blocker amiloride and by treatment with a cAMP analog as well as the CFTR inhibitor 172. Cross-talk among epithelial sodium channel (ENaC) and CFTR has been described in the literature and CFTR is known to regulate other ion channels within the cell. Of note is the observation that ENaC is differently regulated in various organs [29]. Data from various laboratories have shown that ENaC is inhibited by CFTR through incompletely understood mechanisms, suggesting that the spontaneous depolarization we detected could be associated to the loss of CFTR inhibitory signal in CF monocytes leading to the hyperactivation of ENaC channels [29]. This interpretation is supported by the effect of amiloride that completely prevents the depolarization recorded in untreated CF monocytes. The inhibition of spontaneous depolarization is also achieved by treatment of monocytes with a cAMP analog. In this case inhibition of ENaC and/or other sodium channels might occur through CFTR-independent mechanisms whose detailed characterization was beyond the scope of this study. Loss of increased sodium absorption is considered very important in humans for the pathogenesis of CF disease as well as for diagnosis using sweat test (ST) and NPD. However this defect was not detected in newborn porcine cystic fibrosis airway epithelia [30]. It would be interesting to test monocytes also in this very recent CF animal model.

The extents of activation of normal monocytes coupled to the loss of response by CF monocytes allow devising an index able to distinguish between CF and non-CF subjects. Of note is the observation that this test might have a unique feature among currently used functional tests such as ST, NPD and ICM, which can discriminate between CF and healthy subjects [31], [32]. In fact the monocytes test appears capable to distinguish also the population of monocytes derived from heterozygous subjects from both normal and CF subjects. Of course this perspective will be further addressed by enrolling more subjects, in particular heterozygous for CFTR mutations even if ROC curve analysis indicate that the difference recorded is unlikely to become of clinical interest. We noticed that genotypes, when grouped according to functional classes, result in similar values of CF-index. This finding suggests that while this test appears capable to identify loss of protein function it fails to discriminate patients according to mutation types. As a relevant case in the presence of 3849+10 kbC>T or 2789+5G>A mutations we have measured comparable CF indexes values as in other nonsense mutations abolishing functional CFTR expression. This case does not detract in any way the potential applications of this test as no other functional test currently employed has the capacity to discriminate among the different genotypes but can only discriminate among healthy and CF individuals. We need to consider, however, the possibility that CFTR expressed in monocytes might be processed differently from epithelia and sweat duct cells. The role of CFTR in macrophages has been reported as a modulator of the inflammatory process in CF (reviewed in [33]). As monocytes represent macrophage precursors present in the bloodstream their role could be different than that of “innocent bystander”, as such monocyte test might target a relevant function involved in the pathogenesis of CF disease. Also differential protein expression levels in various cell types is not unusual in selected cases, such as in individuals carrying a splicing CFTR mutation, 3849+10 kb C→T where the regulation of alternative splice site selection may be an important mechanism underlying partial penetrance in CF [34].

CFTR activity in monocytes may or may not be correlated with CFTR activity in other tissues. Our results in monocytes show concordance with airways epithelial cells by comparison of CF index with Wilschanski's index. In the presence of mutations known to be associated with normal-borderline sweat test (ST) CF index was negative (table 1 and recent unpublished data). However in these cases the absence of correlation of defective CFTR activity in monocytes with CFTR activity in sweat gland cells indicate the potential capability of the monocyte test to correctly identify CF subjects in difficult cases.

These results might have relevant clinical implications as, although the genetic test represents a major improvement in our capability to diagnose CF, ST represents the standard procedure to confirm a CF diagnosis in the presence of CF symptoms/positive CF newborn screening [35], [36]. According to recent guidelines for diagnosis of CF, ST alone may not be utilized to diagnose CF [36]. Even the combination of ST and genetic test is not always sufficient to diagnose CF. Therefore functional tests performed directly on epithelial cells such as NPD (nasal mucosa) or ICM (rectal biopsies) are required for CF diagnosis [37]. The usefulness of functional tests other than ST has been established in recent best practice guidelines [38]. Despite many efforts the issue of standardization of functional assays (NPD and ICM) is still a matter of discussion [19], [21]: efforts to resolve this important issue are currently ongoing within both European Cystic Fibrosis Society and Cystic Fibrosis Foundation. For these reasons, the proposal of a new functional assay capable to complement the above-mentioned ones can be of interest in the field. Another potential application of the assays we describe is in translational research. For instance, as the variables we analyzed targets myeloid cells whose involvement in the pathogenesis of CF is becoming increasingly recognized in the field [9], [39], [40], this approach might represent a valuable marker of the therapeutic effects of CFTR-targeted drugs in CF patients and they might be used to test the efficacy of any drugs targeting mutations affecting CFTR expression (by western blot) or function (by cell membrane depolarization assay), such as the ones currently under clinical evaluation [41]. In this regard, it is of interest to note that the assays described provided CF values (a negative CF-index) in all the cased diagnoses as CF. We demonstrated that in similar experimental condition both nasal epithelial cells and monocytes can be tested for CFTR function in vivo and in vitro, respectively as applied on 21 CF subjects. In addition to its apparent reliability, testified by these results, some unique advantages can be identified for the monocyte test:

1. It can be performed within a few hours after blood collection. In this case, it is likely that cells removed from the blood might better maintain the effects of any drug previously administered to the patient proving evidence of *in vivo* effects.
2. It is easily repeatable with a minimal discomfort and risk for the patient, and could thus allow a time-course evaluation of effects of any particular therapy on CFTR expression or functional activity, whatever is appropriate according to the treatment.

The monocyte assay, once fully validated by independent research centers, could thus provide a significant advantage in ongoing and future clinical trials. Although the data from the literature, the whole body of antibody and mRNA expression data combined with the use of samples derived from CFTR-defective individuals indicate with high confidence the expression and the function of CFTR in human monocytes, the assessment of CFTR expression by antibody-independent techniques might be useful to further enhance the confidence of our findings. Future directions of these studies include the evaluation of this test in various pulmonary diseases, the use of cryopreserved monocytes, of new specific fluorescent probes and the development of an automated reading system that might expedite the analysis.

## Supporting Information

Figure S1 **ROC curve showing the joint variation of sensitivity and specificity at different thresholds of CF-index to discriminate HTZ from non-CF participants.** The ROC curve was drawn computing the sensitivity and specificity in classifying participants as heterozygotes or non-CF according to different CF-index thresholds. Although the AUC is 0.81 (95%CI: 0.67;0.95), the graph reveals the poor ability of CF-index to separate the two groups: none of the thresholds yielded satisfactory values of sensitivity and specificity. Their maximum joint values are 83.9% and 63.6% respectively.
(TIF)

Figure S2 **Scatter-plot of CF-index against Wilschanski index values.** The graph shows the presence of two outliers for which the value of the Wilschanski's index is particularly high, consistently with the responses to low-Cl and isoprotenerol obtained in NPD tracings. The graph shows a clear separation into groups: in the upper-left quadrant defined by CF-index>0 and Wilschanski's index <0.85 lay all the controls, whereas in the bottom-right quadrant lay all the CF participants.
(TIF)
