# Supplementary figures and images for: Defective CFTR Expression and Function Are Detectable in Blood Monocytes: Development of a New Blood Test for Cystic Fibrosis
Source: PLoS One. 2011 Jul 21;6(7):e22212. doi: 10.1371/journal.pone.0022212 (PMC3141019; doi:10.1371/journal.pone.0022212)

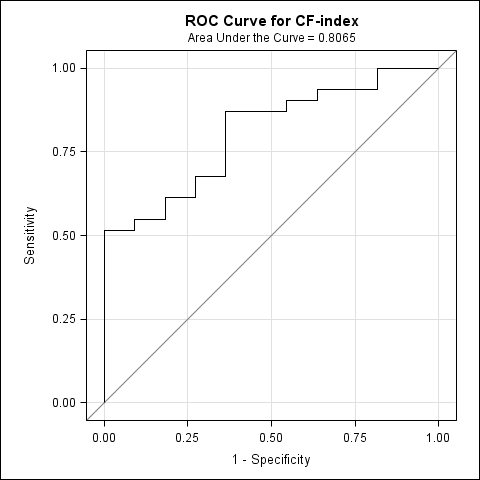

Supplement: Figure S1 — ROC curve showing the joint variation of sensitivity and specificity at different thresholds of CF-index to discriminate HTZ from non-CF participants. The ROC curve was drawn computing the sensitivity and specificity in classifying participants as heterozygotes or non-CF according to different CF-index thresholds. Although the AUC is 0.81 (95%CI: 0.67;0.95), the graph reveals the poor ability of CF-index to separate the two groups: none of the thresholds yielded satisfactory values of sensitivity and specificity. Their maximum joint values are 83.9% and 63.6% respectively. (TIF) [file pone.0022212.s001.tif]

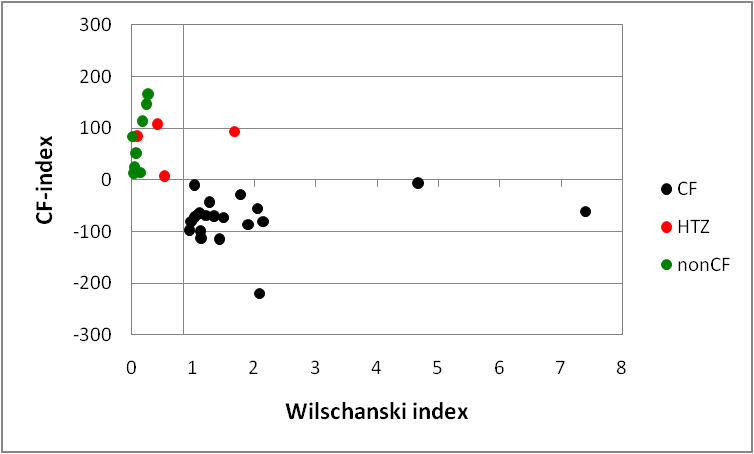

Supplement: Figure S2 — Scatter-plot of CF-index against Wilschanski index values. The graph shows the presence of two outliers for which the value of the Wilschanski's index is particularly high, consistently with the responses to low-Cl and isoprotenerol obtained in NPD tracings. The graph shows a clear separation into groups: in the upper-left quadrant defined by CF-index>0 and Wilschanski's index <0.85 lay all the controls, whereas in the bottom-right quadrant lay all the CF participants. (TIF) [file pone.0022212.s002.tif]
